# Baricitinib statistically significantly reduced COVID-19-related mortality: a systematic review and meta-analysis of five phase III randomized, blinded and placebo-controlled clinical trials

**DOI:** 10.1093/biomethods/bpae002

**Published:** 2024-01-23

**Authors:** Sivananthan Manoharan, Lee Ying Ying

**Affiliations:** Molecular Pathology Unit, Cancer Research Centre, Institute for Medical Research, National Institutes of Health, Ministry of Health Malaysia, Shah Alam, Selangor 40170, Malaysia; Department of Biomedical Sciences, Asia Metropolitan University, Johor Bahru, Johor 81750, Malaysia

**Keywords:** baricitinib, COVID-19, meta-analysis, mortality, randomized controlled trials

## Abstract

Due to high heterogeneity and risk of bias (RoB) found in previously published meta-analysis (MA), a concrete conclusion on the efficacy of baricitinib in reducing mortality in coronavirus disease 2019 (COVID-19) patients was unable to form. Hence, this systematic review and MA were conducted to analyse whether RoB, heterogeneity, and optimal sample size from placebo-controlled randomized controlled trials (RCTs) are still the problems to derive a concrete conclusion.

Search engines PubMed/MEDLINE, ScienceDirect, and other sources like preprints and reference lists were searched with appropriate keywords. The RoB and MA were conducted using RevMan 5.4. The grading of the articles was conducted using the GRADEPro Guideline Development Tool.

Ten RCTs were included in the current systematic review. Only five low RoB articles are Phase III placebo-controlled RCTs with a high certainty level based on the GRADE grading system. For the MA, based on five low RoB articles, baricitinib statistically significantly reduced mortality where the risk ratio (RR) = 0.68 [95% confidence interval (95% CI) 0.56–0.82; *P *<* *0.0001; *I*^2^ = 0%; *P *=* *0.85]. The absolute mortality effect (95% CI) based on the grading system was 35 fewer mortalities per 1000 COVID-19 patients, whereas in the baricitinib and control groups, the mortality was 7.4% and 10.9%, respectively.

With the presence of an optimal sample size of 3944 from five low RoB–placebo-controlled RCTs, which represent a minimum of 300 million population of people and with the presence of 0% heterogeneity from MA, the effectiveness of baricitinib in reducing the mortality in COVID-19 patients is concretely proven.

## Introduction

As of 31 December 2023, approximately 700 million of total coronavirus disease 2019 (COVID-19) cases have been reported worldwide which resulted in more than 6.96 million deaths [1]. The emergence of the severe acute respiratory syndrome corona virus 2(SARS-CoV-2) variants like Alpha, Beta, Gamma, Delta, Omicron, and several more further challenges the healthcare systems. The continuous efforts are being made by scientists to discover new therapies for global citizens who are infected with the SARS-CoV-2 virus. One of the strategies was to repurpose an anti-rheumatoid arthritis drug, baricitinib, for the management of COVID-19 patients. Baricitinib, a Januse Kinase 1 (JAK 1) and 2 (JAK 2) inhibitor is the first immunomodulator, which was found to reduce mortality (death) in COVID-19 patients. Baricitinib was revealed to decrease various cytokines and biomarkers involved in COVID-19 pathophysiology. This drug is available in tablet form and due to its affordability, they are used in low- and middle-income nations [2, 3]. A recent meta-analysis (MA) published in eClinicalMedicine—*The Lancet* which is based on four randomized controlled trials (RCTs) reported that baricitinib statistically significantly reduced mortality in COVID-19 patients [4]. In the MA, the authors have included RECOVERY study-related data where the RECOVERY study was an open-label study which was subject to high risk of bias (RoB). Furthermore, based on the MA for mortality, the heterogeneity (*I*^2^) was significant with *I*^2^ = 65% and *P* = 0.04. When the RoB and heterogeneity are high/significant, the data need to be interpreted carefully. Besides, RECOVERY was conducted in only one country and not representing the world’s population. With the availability of the latest ACTT-4 and several more RCTs data related to mortality, the MA needs to be updated to guide the clinicians with the latest information related to baricitinib for the management of COVID-19 disease. In the current work, the authors updated the systematic review (SR) with the latest mortality data from four RCTs with the aim to derive a clear-cut conclusion for mortality. The MA was only carried out for RCTs with placebo (five studies) study design. We did not pool all 10 RCTs because of the presence of different risks of biases and importantly, pooling different study designs may interfere with conclusion.

## Methodology

In the latest SR and MA, the Preferred Reporting Items for Systematic Reviews and Meta-Analyses (PRISMA) guidelines were followed to shape this review. The guidelines were followed accordingly. No advanced protocol associated with the latest SR and MA was made or registered.

### Research questions

We have formulated three research questions:

1. What is the statistical ability of baricitinib to reduce mortality/death in COVID-19 patients?
2. Do heterogeneity and RoB remain as obstacles to derive a clear-cut conclusion for mortality?
3. Is the sample size enough to make a concrete conclusion?

### Search strategies, article eligibility criteria, and data charting process

Two well-known databases, known as PubMed/MEDLINE, ScienceDirect, and additional sources, such as preprints and reference lists were explored systematically with keywords, specifically ‘Randomised controlled trials baricitinib COVID-19; Randomised controlled trials baricitinib SARS-CoV-2 virus; and Randomised controlled trials baricitinib pneumonia’. The search was done manually in the preprint and reference list. The searched year was between 2020 and 1 December 2023. Additional re-search was done on 31 December 2023 and no new article was found. The inclusion criteria were:

- patients disease-ridden with the SARS-CoV-2 virus;
- baricitinib was used for intervention purposes;
- compulsory existence of proper control/s;
- stringently only for RCT works;
- clinical efficacy mentioned in the study results; and
- language restriction: only English language articles.

The data charting process was undertaken entirely by two authors, independently. Both authors had no issue with any shortlisted articles and thus did not require a third person’s involvement to resolve the problem.

### RoB analysis, grading the evidence using GRADEpro and conduct of MA

The Cochrane RoB tool was used to determine the quality of RCTs [5]. Moreover, the authors conducted an additional step to grade the quality of the included articles with GRADEpro Guideline Development Tool software [6]. The MA was conducted via Review Manager 5.4.1 [5]. Dichotomous data type was selected. The data related to placebo-controlled RCTs were pooled, and relative risk, confidence interval (CI), and Mantel–Haenszel statistical methods were employed. The heterogeneity was evaluated based on the generated *P*-value, the *I*^2^ test. Heterogeneity was considered significant when *P *<* *0.1 or *I*^2^ >50% [7]. A fixed-effect or random-effect model was selected when the data were homogeneous or heterogeneous, respectively. The publication bias (PB) was not studied because only five RCTs–placebo-related articles are available. A minimum of 10 articles are needed to yield a good statistical-related funnel plot to detect PB.

## Results

**Figure 1.**
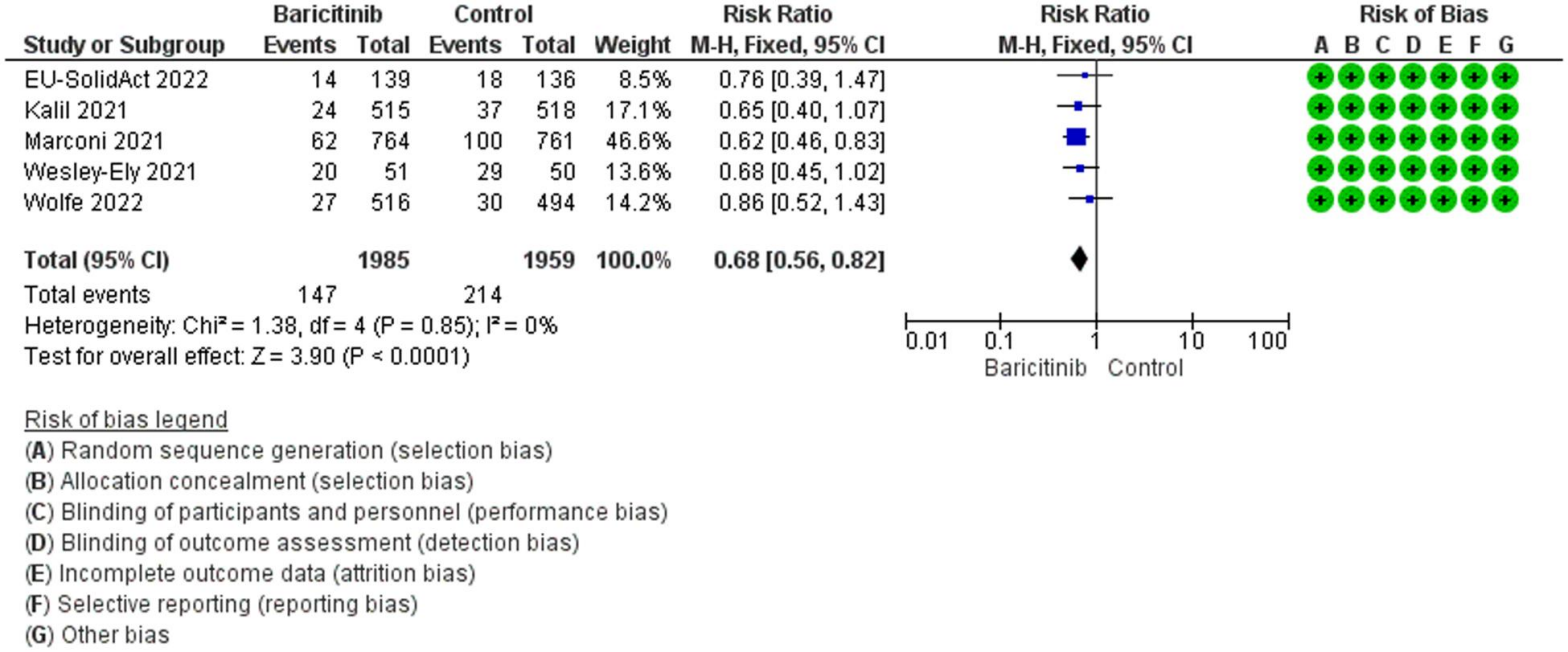
The outcome of baricitinib on mortality from placebo trials only.

Based on the literature search, 10 and 5 RCT articles were included in qualitative and quantitative analyses, respectively. Table 1 was adopted and modified from the authors’ own published MA manuscript in the *Canadian Journal of Infectious Diseases and Medical Microbiology* [8]. Based on Table 1, baricitinib was divided into two doses, which are 2 mg or 4 mg (depending on the health of the patients) and was given to the included COVID-19 participants for a maximum of 14 days. The age of the participants in both groups was consistent in all studies except for two open-labelled studies. However, the age is consistent in all Phase III placebo-controlled RCTs with low RoB. This consistency is important to derive a good conclusion. Although some little variations in genetics may present in the overall study populations (about 0.1%) [9], significant variation in age will be a major confounding factor for MA. By looking at the period of study, most studies started in mid-2020 to late 2020 and concluded in 2021 with two studies completed in March and May 2022. During this period, different variants of the SARS-CoV-2 virus emerged and the authors of the RCTs did not mention which strain of viruses they were looking at. We think that there could be more than one strain present in most (or all) of the studies. This is because most of the SARS-CoV-2 strains emerged in 2020 with Omicron in 2021 [10]. Out of 10 RCTs, 5 of them were categorized as low RoB. Five RCTs with open-label type of study were revealed to have high RoB. Based on grading the evidence in Table 2, only five low RoB–RCTs–placebo articles were included and produced high certainty of evidence. High certainty data will lead to high confidence in the output of MA. Based on the grading analysis, it is revealed that all five studies not only have low RoB but are also not serious in terms of inconsistency, indirectness, and imprecision. All these criteria are not only increasing the certainty level but also will lead to high quality of MA output which can guide clinicians and policymakers for future events or discussions. Besides, based on this grading methods, the absolute effect of baricitinib can be derived, where, based on the inclusion of five low RoB and placebo-controlled studies, the absolute effect was 35 fewer mortalities per 1000 COVID-19 patients treated with baricitinib. In Fig. 1 of MA, the heterogeneity was low with *I*^2^ = 0% and *P *=* *0.85. A fixed effect model was selected for the analysis. Treatment with baricitinib statistically significantly reduced mortality with the risk ratio (RR) = 0.68 (95% CI 0.56–0.82; *P *<* *0.0001). Based on the calculated optimal information size (OIS) for Fig. 1 (data not shown), the inclusion of five low RoB studies provided optimal information to derive conclusions. The total 3944 sample size in Fig. 1 yielded the required OIS. In fact, according to Eli Lilly, approximately 1 million patients received baricitinib for the management of COVID-19 disease in 15 countries [12]. With a margin of error of 2.5% and confidence = 95%, the sample size of 3944 represents a population of a minimum of 300 million [13]. Based on Table 1, three out of five low RoB studies have outcomes where reduction in mortality is not statistically achieved. The data were pooled and the MA was conducted for these three studies (ACTT-2, ACTT-4, and Bari/EU SolidAct). Despite 0% heterogeneity and with the presence of optimal sample size (2318 participants or almost 59% of the overall sample size of 3944), indeed, the statistical significance was not achieved where the RR = 0.75 (95% CI 0.55–1.02; *P *=* *0.07; *I*^2^ = 0%; *P *=* *0.74). When the data of the remaining two studies were pooled, the statistical significance was achieved where the RR = 0.63 (95% CI 0.49–0.81; *P *=* *0.0003; *I*^2^ = 0%; *P *=* *0.72) (MA images are not shown). In fact, these two studies have the required optimal sample size (1626 participants) and produced 0% heterogeneity, too. A minimum sample size of 1537 with a margin error of 2.5% and confidence = 95% is required to represent 300 million people [13]. When pooling all five studies together, a much better statistical significance was achieved (*P *<* *0.0001). This shows several placebos-controlled RCTs need to be conducted to derive more meaningful outcomes. Depending on just one or two RCTs to make conclusion probably led to less meaningful outcome and in worse scenario could cause cessation of that investigation for COVID-19 patients.

**Table 1.** Characteristics of the included studies

Study	Study design and phase	Period of study	Country	Population and age (B versus C)	Intervention	Comparator	Outcomes^a^	Cochrane RoB
RECOVERY [15]	RCT-open label and platform trial (factorial design)	February 21 to December 21	One country	>100 centres/hospitals (58.5 versus 57.7)	4 mg baricitinib for 10 days Reduced dose in case of low eGFR (<60 ml/min) or patients taking probenecid or in children <9 year old	Usual care	Mortality: Reduced	**High** (due to open-label study design)
ACTT-2 [16]	Full RCT and phase III trial	May to July 20	Eight countries	67 centres (55 versus 55.8)	Baricitinib 4 mg (maximum 14 days) with remdesivir (maximum 10 days) Baricitinib 2 mg (with other health related problems) (maximum 14 days) with remdesivir (maximum 10 days)	**Placebo** with remdesivir	Mortality: Not reduced	**Low**
COV-BARRIER [17]	Full RCT and Phase III trial	June 20 to January 21	12 countries	>100 centres (57.8 versus 57.5)	Baricitinib 2 mg or 4 mg with matching SOC for maximum 14 days	**Placebo** with SOC	Mortality: Reduced	**Low**
COV-BARRIER SEVERE [18]	Full RCT and Phase III trial	December 20 to April 21	Four countries	18 centres (58.4 versus 58.8)	Baricitinib 2 mg or 4 mg with SOC for maximum 14 days	**Placebo** with SOC	Mortality: Reduced	**Low**
ACTT-4 [19]	Full RCT and Phase III trial	December 20 to April 21	Five countries	67 centres (58.2 versus 58.5)	Baricitinib (4 mg or reduced dose in case of low eGFR for 14 days/death) plus remdesivir (200 mg loading dose then 100 mg maintenance dose for up to 10 days/discharge/death) plus placebo	Dexamethasone (6 mg for up to 10 days/discharge/death) plus remdesivir plus **placebo**	Mortality: Not reduced	**Low**
Bari/EU-SolidAct [20]	Full RCT and Phase III trial	June 21 to March 22	10 countries	39 centres (59 versus 60)	Baricitinib (4 mg) with SOC for up to 14 days	P**lacebo** with SOC for up to 14 days	Mortality: Not reduced	**Low**
Karampitsakos [21]	RCT-open label	October 21 to May 22	One country	Three 3 centres (73 versus 72)	Baricitinib (4 mg) for up to 14 days or until discharge Baricitinib (2 mg) in low eGFR	Tocilizumab (8 mg/kg i.v) and second infusion within 48 h in case of rapid health deterioration	Mortality: Reduced	**High** (due to open label study design)
PANCOVID/Montejano [22]	RCT-open label and Phase III pragmatic trial	October 20 to September 21	One country	25 centres (68 versus 67)	Baricitinib (4 mg) for 10–14 days with SOC Baricitinib (2 mg) for patients >75-year-old	Dexamethasone (6 mg oral or i.v) for 7–10 days	Mortality: Not reduced	**High** (due to open label study design)
Morales‐Ortega [23]	Exploratory RCT-open label and Phase II with pick‐the‐winner design	September 20 to June 21	One country	One centre (55.5 versus 55.8)	(i) Baricitinib (4 mg q.d., 7 days) plus standard care	Imatinib (400 mg q.d., 7 days) plus standard care Standard care alone	Mortality: Not reduced	**High** (due to open-label study design and pick-the-winner design)
TACTIC-R [25]	RCT-open label and Phase IV trial	May 20 to May 21	One country	22 centres (61.4 versus 62.2)	Baricitinib (4 mg) for up to 14 days or until discharge and the dose was adjusted according to the age	Ravulizumab (5 mg/ml i.v). The dose was adjusted according to weight SOC	Mortality: Not reduced	**High** (due to open label study design)

a The outcomes were based on the statistical significance. The PDF article was provided by corresponding author of Morales‐Ortega [23] after requested through email conversation on 17 May 2023.

IMV, invasive mechanical ventilation; SOC, standard of Care; B, Baricitinib; C, Control; eGFR, estimated glomerular filtration rate; ECMO, extracorporeal membrane oxygenation; NA, not available; i.v, intravenous; q.d., every day.

**Table 2.** Grading the evidence with GRADEpro Guideline Development Tool

Certainty assessment							No. of patients		Effect		Certainty	Importance
No. of studies	Study design	RoB	Inconsistency	Indirectness	Imprecision	Other considerations	**Baricitinib** **(treatment)**	**Placebo** **(control)**	**Relative** **(95% CI)**	**Absolute** **(95% CI)**		
Mortality_5 RCTs_Low RoB_Placebo study designs												
5	Randomized trials	Not serious	Not serious	Not serious	Not serious	None	147/1985 **(7.4%)**	214/1959 (**10.9%)**	**RR 0.68** (0.56 to 0.82)	**35 fewer per 1,000** (from 48 fewer to 20 fewer)	**⊕⊕⊕⊕** **High**	CRITICAL

## Discussion

In the previous work by Selvaraj *et al*. [4], the authors grouped the RCTs without taking into consideration about the included study designs and RoB of each study. Pooling RECOVERY [15] together made the results unreliable because this study has a high RoB which includes open-labelled trial design. Furthermore, RECOVERY [15] alone had 8000 plus study participants and accounted for 40.2% of the weightage in the overall MA conducted by Selvaraj *et al*. [4], which could probably mask the actual outcomes from the placebo-controlled trials. Unlike placebo-controlled trials, in RECOVERY [15], the participants came from only one country which further reduced the reliability of the MA conducted by Selvaraj *et al*. [4]. In current work, although all baricitinib RCTs-related studies are included in the SR, only placebo-controlled trials are included for MA. The exclusion of open-labelled studies is largely due to both drug administrator and study participants being alert of the drug and treatment given [11]. This will lead to bias in the outcome of the results. Besides, in all five open-labelled studies, the studies were not conducted globally but only focused in one country. This is not the case for placebo-controlled trials. It is important to conduct studies globally in Phase III trials because Asians could have a non-similar outcome to certain medicines. The results from trials, which are carried out in non-Asian patients probably may not always be applicable to Asian peoples. Scientists should take into consideration the number of Asian participants in future studies before generalizing the results for any Asian populations [26]. Blinded placebo-controlled RCT are more powerful than non-blinded and/or non-placebo RCT because this study design is a gold standard for clinical trials. For example, several open-labelled studies have shown that certain medicines are beneficial for disease X, but it has shown the opposite effect based on larger placebo-controlled trials. Baricitinib is an anti-rheumatoid drug and repurposed for COVID-19 therapy. Although baricitinib is an FDA-approved drug for rheumatoid arthritis, baricitinib is an experimental drug for COVID-19. An experimental drug could be beneficial, non-beneficial, or probably harmful to COVID-19 patients. The advantage of baricitinib is that it has a fully established safety profile in humans before it gets FDA approval for rheumatoid arthritis but remains experimental for COVID-19 therapy until low RoB and placebo-related RCT results are available [27]. Indeed, low RoB and larger placebo-controlled clinical trials with multi-national participation are the key to converting the status of an experimental drug to a clinically functional drug for COVID-19 therapy. In terms of the age of the participants, in all five low RoB placebo-controlled trials, the age range was between 55- and 60-year-old. According to Peng *et al*. [28], the authors described the mean age 54.21 as middle-aged adults, while 67.68 as old age. Based on this understanding, we speculate that the findings from all five low RoB articles are representing middle-aged to late middle-aged adults. The efficacy of baricitinib in the much older population is little known. On 1 December 2022, ABC News reported that above 90% of deaths related to COVID-19 happened among old adults [29]. Americans aged ≥65 years composed 92% of all deaths resulted from the SARS-CoV-2 virus. The ABC News mentioned that it is no doubt that age plays a huge role as the most influential risk factor for COVID-related mortality [29]. Despite the small age range gap between all five low RoB studies, three out of five studies have outcomes where reduction in mortality is not statistically achieved. There are many factors governing these outcomes, such as the study sites (or country), ethnicity, the gender of the participants, the number and health state of older populations in the trials, the underlying illness, COVID-19 disease severity, and several more. In certain studies, male participants contribute higher percentages than females, while in a few more studies the proportion is almost equal. In some studies, there are Asians approximately 7–13% but in a few studies no Asians or very small percentages are spotted. Different rates of obesity, diabetes, hypertension, cholesterol, autoimmune disorders (co-morbid) too were spotted from one study to another. These could play roles in the overall outcomes of the study. In fact, there could be Epstein–Barr virus (EBV) reactivation in COVID-19 patients which further affects the outcomes of the study. It has been shown that EBV reactivation in hospitalized COVID-19 patients had a statistically significantly higher mortality rate (23.6%) than EBV(−)/SARS-CoV-2(+) patients (9.9%). Furthermore, the absolute mortality outcome because of EBV reactivation was 130 more per 1000 COVID-19 patients. The authors too suggested that EBV reactivation must be suspected since EBV reactivation is likely an indication for COVID-19 disease severity [30]. Based on these arguments, it is possible that hospitalized COVID-19 patients probably die due to the infection of more than one virus. In our opinion, the MA for EBV reactivation was based on small number of sample size (because limited availability of studies), and these data need to be analysed and read cautiously but we never ignored the involvement of EBV reactivation and related outcomes in terms of mortality in COVID-19 patients. Despite the outcomes of the individual studies, in the current MA, when the data from five low RoB placebo-controlled studies were pooled together, we found that baricitinib statistically significantly reduced mortality in COVID-19 patients. Based on Fig. 1, with the presence of optimal sample size of 3944 from five low RoB–placebo-controlled studies which represents a minimum of 300 million population of people, the effectiveness of 2 mg or 4 mg baricitinib in reducing the mortality in COVID-19 patients is concretely proven. In conclusion, the availability of five low RoB–RCTs–placebo-related articles helped to solve the high heterogeneity and RoB issues found in the previously published article to derive a concrete conclusion on the effectiveness of baricitinib in reducing mortality in COVID-19 patients.

## Supplementary Material

bpae002_Supplementary_Data

## Data Availability

The authors used published primary data that are available online.
