# Supplementary material for: Baricitinib statistically significantly reduced COVID-19-related mortality: a systematic review and meta-analysis of five phase III randomized, blinded and placebo-controlled clinical trials
Source: Biol Methods Protoc. 2024 Jan 23;9(1):bpae002. doi: 10.1093/biomethods/bpae002 (PMC10873572; doi:10.1093/biomethods/bpae002)
Supplement: bpae002_Supplementary_Data [file bpae002_supplementary_data.doc]

**Screening**

**Included**

**Eligibility**

**Identification**

Records identified from 2 databases PubMed/MEDLINE and ScienceDirect.

**(n=157)**

Records after duplicates removed
(n=83)

Records screened
(n=83)

Full-text articles assessed for eligibility
(n=57)

Records included in qualitative synthesis
**(n=10)**

Records included in quantitative synthesis (meta-analysis)
**(n=5)**

Additional records identified through preprints and reference list search. Strictly searched for related research articles only
**(n=18)**

Records excluded (Meta-analysis and abstract)
(n=26)

Full-text articles excluded, with reasons (n=47)

**Supplementary file S1:** PRISMA flow chart of articles inclusion and exclusion
